# How Human Resources Index, Relational Justice, and Perceived Productivity Change after Reorganization at a Hospital in Sweden That Uses a Structured Support Model for Systematic Work Environment Management

**DOI:** 10.3390/ijerph182111611

**Published:** 2021-11-04

**Authors:** Erebouni Arakelian, Sofia Paulsson, Fredrik Molin, Magnus Svartengren

**Affiliations:** 1 Department of Surgical Sciences, Uppsala University, 752 37 Uppsala, Sweden; 2 Department of Medical Sciences, Occupational and Environmental Medicine, Uppsala University, 752 37 Uppsala, Sweden; Sofia.Paulsson@hpihealth.se (S.P.); fredrik.molin@ipf.se (F.M.); magnus.svartengren@medsci.uu.se (M.S.); 3 IPF, The Institute for Organizational and Leadership Development, Uppsala University, 753 20 Uppsala, Sweden

**Keywords:** hospital setting, work environment, intervention, participative, productivity

## Abstract

To facilitate systematic work environment management, which should be a natural part of business development, a structured support model was developed. The Stamina model has previously been used in Swedish municipalities, showing positive results. The aim was to study how the Human Resources Index (HRI), relational justice, short-term recovery and perceived productivity changed in a recently reorganised perioperative setting in a hospital in Sweden that uses a structured support model for systematic work environment management. A longitudinal design that took measurements at four time points was used in a sample of 500 employees in a perioperative hospital department. The results for the overall sample indicated a positive trend in the HRI (Mt1 = 48.5, SDt1 = 22.5; Mt3 = 56.7, SDt1 = 21.2; *p* < 0.001). Perceived health-related production loss (Mdt1 = 2, IQR = 3; Mdt3 = 0, IQR = 3; *p* < 0.001) and perceived work environment-related production loss (Mdt1 = 2, IQR = 3; Mdt3 = 0, IQR = 4; *p* < 0.001) showed major improvements. Short-term recovery showed a minor improvement (Mt1 = 2.61, SDt1 = 1.33; Mt3 = 2.65, SDt3 = 1.22; *p* = 0.872). In conclusion, the implementation of the Stamina model, of which the HRI constitutes an important part, seems to be a helpful tool to follow-up on work environment processes, and minimise production losses due to health and work environment-related issues.

## 1. Introduction

The work environment refers to biological, medical, physiological, psychological, social and technical factors that affect the individual in the work situation or in the workplace environment. There are incentives to improve the work environment, including the need to reduce the risk of accidents and death, reduce the risk of work-related illnesses, and to promote the health and productivity of employees. Work places that are developed may also focus more on improving conditions, rather than avoidance of risks only. In short, they aim to create conditions that are as good as possible for carrying out work in a safe and efficient manner. In many parts of the world, there is legislation regarding occupational health and safety in order to protect the workforce from poor working conditions. According to Swedish legislation, employers are obligated to work systematically on environment issues in the workplace, including both physical and psychosocial aspects (AFS 2001:1) [1,2]. This means that employers are obligated to assess risks and take actions to improve the work environment and follow-up the cycle annually. Studies indicate that many workplaces in Sweden do not meet the requirements for systematic work environment management as intended by the legislation [3]. There are many reasons for this, with lack of time being one of them [4]. To evaluate and systematically improve work environment factors in a more actionable way, a Structured and Time-effective Approach through Methods for an Inclusive and Active working life was created; this is also referred to as the Stamina model. The model provides structured and continuous feedback and group reflection in the process of systematic work environment management, [5] with a built-in process feedback measurement called the Human Resources Index (HRI) [5]. In this study, the model was implemented in a perioperative setting in a Swedish hospital as a continuation of a larger project performed in Swedish municipalities.

### 1.1. Aim

The aim was to study how the Human Resources Index, relational justice, short-term recovery and perceived productivity changed in a recently reorganised perioperative setting in a hospital in Sweden that uses a structured support model for systematic work environment management.

### 1.2. Background

#### 1.2.1. Employee Health in Relation to Organisational and Relational Justice, Productivity and Recovery

Employee health may be affected by several factors in the work environment. Regarding the organisational and social work environment, factors such as job demand, job control, and perceived fairness in the organisation have proven important, and these are partly mediated by sleep quality and short-term recovery [6,7]. In turn, employee health affects work-related productivity loss [1,8,9].

The Demand Control Support Model of Karasek and Theorell [1] explains how employee health is positively related to feelings of job control and to social support in the workplace, and negatively influenced by high work demands [10,11]. Studies suggest that first-line managers play an important role in supporting their employees, which improves well-being [12], job satisfaction [13] and the employees’ engagement at work [1,14]. However, first-line managers often feel uncertain about how to engage and support their employees [15], which is why tools are needed to address this [15,16].

Another aspect of the social work environment is perceived fairness in the organisation, often referred to as organisational justice. Organisational justice is divided into four dimensions: distributive justice, procedural justice, interpersonal justice and informative justice [8]. In the present study, the focus is on interpersonal justice (also referred to as relational justice or interactional justice), which emphasises the superior’s relationship to the employees, for example, how the senior manager handles employees’ personal views and rights, and if the employees are treated impartially, truthfully and with kindness. Thus, relational justice relates to being treated fairly at the workplace and it has been shown to be linked with health in the workplace [17,18,19,20,21]. This refers to how the superior managers treat their employees [19].

In a series of studies by Finnish researchers, the connection between organisational justice and several health-related parameters such as sickness absence [22], long-term inflammatory markers [23], smoking [24] and alcohol consumption [25] was examined. The results indicated a relationship between mental illness and relational justice and procedural justice [8,17]. Furthermore, studies show that how fairly employees are treated can predict future ill health [22], self-rated health and burnout [26], and metabolic syndrome [27]. It has been shown that perceived leadership and cardiovascular disease [28] are interconnected. Also, it has been shown that employees in companies with a higher justice index had higher ratings for their health and well-being [18,19]. Moreover, employee well-being is affected by leadership, social climate and commitment [29]. The model for organisational justice has conceptual connections to the above-mentioned demand control support model [8].

Low levels of organisational justice have been indicated to affect both men and women, resulting in sleeping problems and sleep onset problems [30]. Quality of sleep, i.e., duration and continuity of sleep, is important for the employees’ recovery. A study by Elovainio et al. [6] concluded that sleeping problems are one of the underlying factors that cause the adverse health effects related to low organisational justice at work among hospital employees. Negative associations have been shown between stress and quality of sleep [31,32], including that insufficient sleep more than tripled the risk for metabolic syndrome and the risk of coronary heart disease [31]. Åkerstedt et al. [32] studied work-related mental strain, and emphasised that sleep disturbances were associated with a higher risk of subsequent long-term sickness absence. In a review study, Linton et al. [7] reported that the psychosocial work variables of social support at work, job control and organisational justice were related to fewer sleep disturbances, while high work demands, job strain, bullying and effort–reward imbalance were related to more future sleep disturbances.

Thus, much is known about how factors in the work environment affect health and productivity, and interventions in this area are highly interesting. Lohela-Karlsson et al. [33] suggest that perceived work environment-related production loss may be used to evaluate the effects of organisational interventions. Productivity loss goes beyond injuries and sickness absence. Work environment-related production is also included. They showed that fair leadership, good social climate in the workplace, role clarity and control of decision had a significant impact on levels of self-reported production loss and that employees who experienced inequality and high demands on making decisions reported significantly higher levels of production loss. This can be interpreted as meaning that fair leadership seems to be a buffering factor that prevents work environment problems from causing production loss. Furthermore, factors linked to the work environment seem to have a greater impact on production loss than factors linked to health [33]. Accordingly, developing and maintaining good health and productivity in business requires a structured process that supports the development of good working environments [34].

#### 1.2.2. The Stamina Model

The Stamina model is a structured participatory support model for systematic work environment management with a built-in process feedback measurement called the Human Resources Index (HRI). HRI can be measured at any given point in time as a single measure, and changes in its value can be used to evaluate how the work environment changes over time [35]. Thus, the organisation can use HRI as a support measure to assess, evaluate and follow-up on the continuous work environment management as required by the law. The Stamina model engages employees and their leaders to manage their work environment in a structured and systematic manner. Four steps form the basis for working with the model [5]. In the first step, a web-based anonymous questionnaire is sent to all employees. The questionnaire contains an open-ended question about how employees perceive their work environment right now. This step aims to anonymously identify relevant work environmental issues. In the second step, a workshop is held as part of a regular workplace meeting, where employees and their managers meet in groups of 8–20 to consider each other’s viewpoints (based on their written answers). Together, the group discusses and evaluates the various work environment factors in what can be described as a type of risk assessment. In the third step, the group decides what actions can be taken to create the desired work situation. In the last step, the work group prioritises one activity to focus on and creates an action plan. Steps one to four create a session, which, according to the model, should be repeated four times annually (every three months). Between the sessions, the groups of employees are given an opportunity to work with their action plans; thereafter, in the next workshop, the action plans are re-evaluated and followed up. The model can be modified and adapted to fit one’s organisational needs. The important cornerstones of the model are structure, recurrent feedback and employee participation. A software solution supports the model at every step [5].

#### 1.2.3. The Stamina Model in Swedish Municipalities and in Perioperative Settings

Results from a recent study on Swedish municipalities using the Stamina model as their support model for approximately 6400 employees showed a positive association between the Human Resources Index (HRI) and relational justice, a positive association between HRI and short-term recovery, a negative association between HRI and work environment-related production loss, and a negative association between HRI and health-related production loss [35]. The authors indicated that monitoring changes in HRI as feedback of the process is a possible way to determine production loss, perceived leadership and short-term recovery in a work group. Data were presented at baseline, not longitudinally. The authors have reported several positive experiences resulting from the use of the model in municipalities in Sweden [36,37], which showed a shift in focus from an individual to an organisational perspective of work. They have also reported that communication and increased understanding of one’s work tasks changed over time, thus contributing to a deeper focus on the actual operation [37].

However, it has been suggested that interventions such as implementation of the Stamina model, might work differently in different contexts [38] since the inner or outer settings, the individuals and the implementation process may vary [39]. This study therefore investigated what happened when the Stamina model was implemented in a new context, namely, in a perioperative setting (i.e., the anaesthesia and operating rooms) in a public hospital in Sweden. The implementation process began one month after a reorganisation of the relevant departments. The working conditions in the operating rooms are unique as these are closed rooms, sometimes without access to daylight. Specialist nurses, i.e., nurse anaesthetists and operating room nurses, often do not leave the room during ongoing surgery due to patient safety and hygiene reasons. Only short lunch breaks of 30–45 min and two short coffee breaks of 10–15 min each are taken by the specialist nurses during day shifts. Operating room nurses usually take their breaks in between appointments for patients. These working conditions and long hours of work place high demands on both nurse anaesthetists and operating room nurses [40] who stay with the patient in the operating room for the entire surgery. Anaesthesiologists are often responsible for more than one patient. They are usually outside the operating rooms and can be contacted when needed by the nurse anaesthetists who are in the operating room with the patient. In case of sickness and sick leave, both short- and long-term employees are asked to move between the different departments within the same organisation, i.e., the perioperative settings. Employees also move of their own free will to increase their competence at work. A study by Walinder et al. [41] on 1400 operating room staff from seven Swedish hospitals reported that lower social support scores and high demands together with low control (high-strain) scores were related to lower well-being, lower zest for work, and more thoughts about leaving the position. Anaesthetists scored in the low-strain field, nurse anaesthetists and assistant nurses in the passive field, and operating nurses in the active field, in comparison to all personnel. A published qualitative study from the perioperative environment described how the change in attitude was first met by skepticism, which subsequently turned into a positive attitude when it was recognised that the Stamina model offers increased participation [42].

Organisational changes, which are common in the public sector in Sweden [43], often have negative consequences, not only on employees’ health, but also on how they experience the work situation [44,45] and the quality of the operational business [46,47,48]. In the European survey, Eurofound 2017, employees who had experienced reorganisations reported higher work intensity, lower self-commitment, more unfair treatment and lack of time to carry out their work tasks. Organisational changes often increase sick leave [49], as well as thoughts of leaving the job [48,50]. Studies have shown that these negative effects can be counteracted by factors such as positive leadership, participation and commitment in the planning of the change [51].

In this study, we wanted to investigate the effect of the Stamina model in a new perioperative setting in a longitudinal manner, using the same outcome measurements from the previous study on the Swedish municipalities (short-term recovery, perceived productivity, organisational justice, HRI and the same design), where the process in the group was the focus, rather than the individuals within the group.

## 2. Materials and Methods

### 2.1. Design

In this study, we studied the effect of the Stamina model in a new perioperative setting in a longitudinal manner, using the same outcome measurements from the previous study on the Swedish municipalities (short-term recovery, perceived productivity, organisational justice, HRI and the same design), with a focus on the process in the group, rather than the individuals within the group.

### 2.2. Sample

A total of 755 aggregated web responses from 500 employees in perioperative settings in a university hospital in Sweden over a period of sixteen months were included. The staff members comprised approximately 140 anaesthetist nurses, 90 operating room nurses, 40 anaesthesiologists, 230 nurse assistants, nurses and assistant nurses from postoperative ward, and managers from different levels in the organisation.

There were approximately 250 employees working in the anaesthesia and operating rooms in the perioperative setting. In connection with a reorganisation in Spring of 2018, approximately 250 employees from other parts of the hospital were included in the anaesthesia and operating room organisational structure at the beginning of 2018. The Stamina model [52] was introduced to approximately 500 employees during February 2018 to improve the work environment in perioperative settings. A few months after the introduction of the model, a reminder meeting was held with a further introduction to the model to strengthen the implementation process. The participating departments operating within the anaesthesia and operating room organisational structure were followed as one group.

### 2.3. Data Collection

The Stamina workshops were held in Autumn of 2018 (t1, *n* = 267), Spring of 2019 (t2, *n* = 214), Autumn of 2019 (t3, *n* = 225) and Spring of 2020 (t4, *n* = 49). Due to the COVID-19 pandemic and the need for changes in the workload, many operating wards could not conduct their Stamina workshops; thus, there are notably fewer participants in the fourth measurement. The results from t4 are reported in the tables but disregarded in the statistical analysis. This omission is explained in detail in the discussion section.

### 2.4. The Online Questionnaire

The online questionnaire in the Stamina model consists of two parts. Part one contains an open-ended question asking the employees to explain what characterises their work environment, and to grade (1) if the experience is positive or negative and (2) perception of opportunity to influence the situation or their “problem” (that they have described). Part two contains questions about health-related production loss, organizational and relational justice, short-term recovery, i.e., feeling refreshed when waking up. Only the open-ended question was shared and discussed in the workshop. The questions in the second part were only used in to evaluate the results of the process.

#### 2.4.1. Health and Work Environment-Related Production Loss

Perceived productivity captures the effect of (1) health-related problems and (2) work environment-related problems on employees’ work performance [9,33,53]. The two questions measuring health-related and work environment- related productivity were formulated as follows: “Over the past 7 days, have you experienced health-related/work environment-related problems at work? Health problems refer to all possible physical and emotional problems or symptoms. In case of a positive answer, a follow-up question was asked, “During the past 7 days, how much did your health-related problems/work environment-related problems affect your performance while you were working?” The first question is answered with yes or no, and the follow-up question can be scored from a scale of 1 to 10, where 1 means “The health-related problems/work environment-related problems had no effect on my work” and 10 means “The health problems/work environment problems completely prevented me from working”. High values represent a large negative impact on performance.

#### 2.4.2. Organisational and Relational Justice (Fairness in the Organisation) Index (RJI)

The Stamina questionnaire includes six statements that are used to measure how senior managers handle the employees’ personal views and rights, and whether the employees are treated impartially, truthfully and with kindness. The statements can be found in the study by Molin et al. [35]. These are converted into a relational justice index (RJI). The response scale contains five levels from 1 to 5, where 1 means does not agree at all, and 5 means agree fully. The RJI score can range from 6 to 30 points, where high values indicate good organisational justice. The index has been shown to be linked to psychosocial outcomes [22,54].

#### 2.4.3. Human Resources Index (HRI)

The Human Resources Index (HRI), which is expressed on a scale from 0 to 100, measures employees’ perceptions of their current work situation. The HRI predicts the risk of adverse health outcomes [53]. It is calculated based on open answers from each participant, depending on what characterises the participant’s current work situation, i.e., whether the experience is positive or negative or to grade the opportunity to influence the situation. High HRI values, >50 indicate a good organisational work environment, whereas low HRI values, <50 indicates a poor work environment. Similar measures have been used previously to study attitude and influence at work [53].

#### 2.4.4. Short-Term Recovery, Feeling Refreshed When Waking Up

One question from the Karolinska sleep form (KSQ) was chosen to assess how well the employees slept [54,55], and to estimate their recovery in the short-term. The question asked was, “Have you experienced any of the following complaints in the past three months: not feeling refreshed when waking up” (KSQ: Question 9). The answers are given on a six-point scale from 1 (meaning never) to 6 (meaning always). The scale is then reversed to indicate how refreshed one feels upon waking up, which results in 1 = never refreshed when waking up, indicating poor recovery to 6 = always refreshed when waking up, indicating a good recovery. This measure was chosen as recovery is a prevents stress-related ill health, and an interactive process between individual experience and the environment [56].

### 2.5. Data Analysis

Data were analysed using R Studio [57]. The descriptive statistics present the mean (M), standard deviation (SD), median (Md), and interquartile range (IQR) for the variables. The significance level was set at *p* < 0.05. The comparison between the time points was calculated using the Student’s *t*-test for HRI and Wilcoxon rank sum test for categorical variables. All operating departments within the anaesthesia and operating room organisational structure in the university hospital included in this study were seen as a whole, or as one group. Individual respondents were not tracked over time, and there is a possibility that different people from the two sub-departments participated over time. No test of difference was conducted at time point four (see above for comment). The relational justice index was calculated as a summation index of the six items measuring relational justice. Cohen’s d was used to calculate the effect size.

## 3. Results

Table 1a,b shows the descriptive statistics for the variables: the Human Resources Index, relational justice index, short-term recovery, health-related production loss, and work environment related production loss at four different time points (t1 to t4). The HRI improved significantly between time point 1 and time point 3 (Mt1 = 48.5, SDt1 = 22.5; Mt3 = 56.7, SDt1 = 21.2; *p* < 0.001). Short-term recovery showed a minor improvement between time point 1 and time point 3 (Mt1 = 2.61, SDt1 = 1.33; Mt3 = 2.65, SDt3 = 1.22; *p* = 0.872). Health related production loss showed an improvement (Mdt1 = 2, IQR = 3; Mdt3 = 0, IQR = 3; *p* < 0.001) as did work environment-related production loss (Mdt1 = 2, IQR = 3; Mdt3 = 0, IQR = 4; *p* < 0.001). Since one item was missing, the relational justice index was not calculated for time point 1 and time point 2.

Table 2 presents the proportions of respondents reporting a HRI below 50.0 is presented in Table 2. The proportion of respondents with HRI below 50.0 dropped significantly from 55.4% to 32.9% between time point 1 and time point 3 (*p* < 0.001). Proportions of respondents reporting health-related production loss and work environment-related production loss dropped from 46.0% to 38.7% and from 52.4% to 49.3%, respectively (*p* = 0.552; *p* = 0.118).

A closer look at relational justice and work environment-related production loss is presented in Table 3 and Table 4. Table 3 shows the effect sizes (Cohen’s d) for having reported work environment-related production loss for the variables HRI, short-term recovery, and for the six statements of relational justice, at time point 1 (one of the items for relational justice was excluded from the analysis at t1; hence the N.A.). Table 4 shows the same variables at time point 3. The effect size on HRI for not having reported work environment-related production loss was large (d = 0.816) at time point 1 and medium (d = 0.516) at time point 3. Effect sizes for the relational justice items were small (0.200 ≤ d ≤ 0.467) at time point 1 as well as for time point 4 (0.296 ≤ d ≤ 0.380). Effect size for short-term recovery was small (d = 0.233) at time point 1 and medium (d = 0.530) at time point 3.

**Figure 1:**
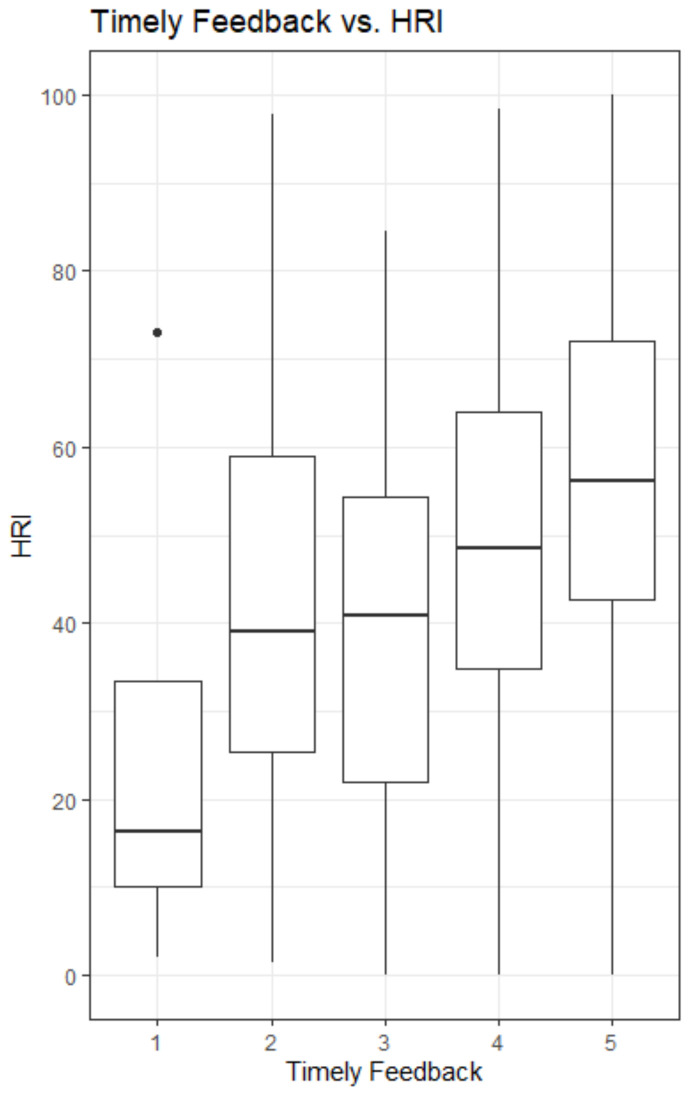
In the overall sample (*n* = 755), respondents who did not receive timely feedback from their supervisor had a lower HRI than respondents who did receive timely feedback (md = 16.4 for strongly disagree and md = 56.2 for strongly agree, answer options: 1 = strongly disagree; 5 = strongly agree).

**Figure 2:**
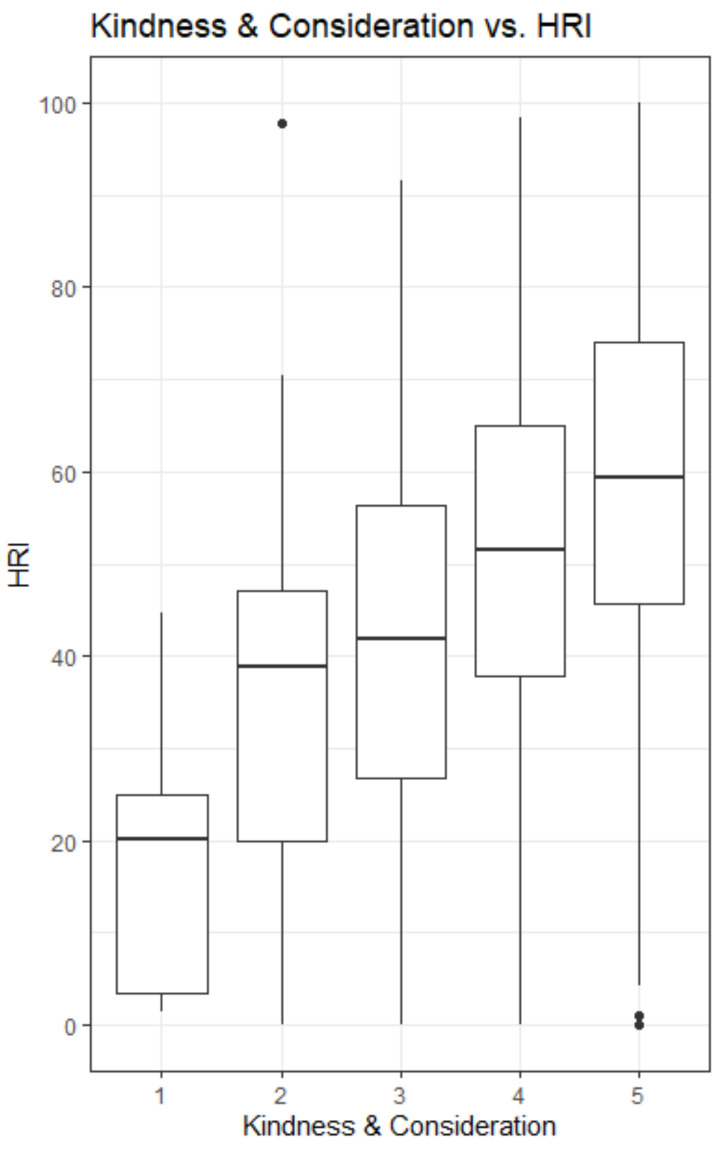
In the overall sample (*n* = 755), respondents who were not being treated with kindness and consideration by one’s supervisor had a lower HRI than respondents who were being treated with kindness and consideration by one’s supervisor (md = 20.1 and md = 59.3).

Figure 1 and Figure 2 show the responses in the overall observations (*n* = 755) for two of the relational justice statements and its relation to the HRI. Those two statements were shown to be the ones that were most clearly related to the HRI-measure. The statements are: “Your supervisor provided you with timely feedback about the decisions and their implications” (Figure 1) and “Your supervisor treated you with kindness and consideration” (Figure 2) (the answer options range from 1 = strongly disagree to 5 = strongly agree). The figures show that those who did not receive timely feedback from their supervisor had a lower HRI than respondents who did receive timely feedback (Md = 16.4 for strongly disagree and Md = 56.2 for strongly agree). This was also true for the statement about being treated with kindness and consideration by one’s supervisor (Md = 20.1 and Md = 59.3).

## 4. Discussion

This study aimed to investigate the effect of the Stamina model in perioperative settings in a longitudinal manner, using the Human Resources Index, relational justice, short-term recovery, and perceived productivity, where the focus was on the process in the group rather than the individuals within the group.

The results of this longitudinal study show that the HRI-measure, health-related productivity loss and work environment-related productivity loss improved significantly in the work group during the study period. The results also imply covariation between low levels of work environment-related productivity loss, high HRI-values and high levels of relational justice and short-term recovery in the group. An important message here is that in a real-life context, there are clear benefits in using only the single built-in process feedback HRI-measurement, which the whole group can easily follow and understand, rather than using several outcome measures. Furthermore, the fact that the individuals in the group can reflect together and follow a process and the trend in their systematic work environment management leads to employee engagement and a change in the work environment [42], which is more important for the group than analysing single results at a given time point.

A previous cross-sectional study suggests that an improvement in the HRI-measure indicates that changes made in the work environment have had a positive impact on productivity and short-term recovery and are also linked to higher relational justice [35]. This connection is confirmed by the present longitudinal study. Specifically, the present study shows similar results to those found in Swedish municipalities when implementing the Stamina model [36], suggesting that the model can support positive changes in the work environment in different settings.

The positive changes seen in the present study occurred in a workplace that had just been through a reorganisation, which usually has negative consequences, both on employees’ health and on how they experience the work situation [44,45]. This strengthens previous findings that the Stamina model supports positive leadership and employee participation [37,52,58], which are factors that have been proven to counteract the negative effects of reorganisation [29,51]. Here, the improvements in HRI and productivity could possibly be attributed to the reorganisation itself or positive changes in the management team, but none of those factors emerged as important factors in previous qualitative studies in the current working group [52].

In the Stamina model, employee participation is organised in a workshop format [5]. Together, the group concretises the meaning of the somewhat abstract concept “work environment” [28,33,45] and creates a shared platform and understanding of the current work environment, focusing on issues that are important for the present work group. The perspective is thus the group and its development in work environment management or what measures they take to reflect, identify and solve problems together in the group. Moreover, the view is bottom-up not top-down, meaning that it is not the management or HR department that prioritises what measures should be taken. It is not focused on just one individual either, as in many work environment apps that address one certain individual [59]. The model could be linked to the theory of how reflection within the work group facilitates a joint focus on work process, strategies, goals and work environment [60]. Reflection within the group has proven to be an important step towards better team performance, especially in terms of team effectiveness (the team meets the goals and expectations [61] and innovativeness (“the introduction of new ideas and processes that are implemented to improve performance of a team”) [62]. Anselmann and Mulder [63] suggest that team reflection is an underlying mechanism that links leadership and team performance.

Further, the results strengthen the use of the entire relational justice index, rather than the single question components. Unfortunately, due to COVID-19 and unexpected circumstances that were out of our control, we could not follow the change in the full index over time. However, the results imply a positive relationship between high HRI-values and having a supervisor that provides timely feedback about decisions and their implications, as well as a supervisor that treats employees with kindness and consideration. This implies that participants that rate their work environment as positive and feel that they can influence it also rate the management as kind and considerate and feel that they give feedback on time. This is well in line with previous results from a qualitative study from the present workplace, which reported that feedback is one of the important factors for the successful implementation of the model [52]. The results also suggest that there is a connection between experiencing that one has a manager who takes one’s viewpoint into consideration and experiencing that one’s productivity is not affected by factors in the work environment. These results strengthen Lohela-Karlsson et al.’s previous theory that fair leadership reduces the effects of work environment factors on productivity [33].

In the current study, short-term recovery was measured by how refreshed you feel upon waking up. The results show a connection between high levels of work environment-related production loss and a feeling of not being recovered, especially at the third measurement point. This strengthens the previous results that propose that sleeping problems and not feeling recovered could be the link between work environmental problems (such as stress), adverse health effects [6,7,31,32] and a higher risk of long-term sickness absence [32].

## 5. Limitations

The study takes place in a complex and specific workplace involving perioperative settings in a Swedish hospital; thus, generalisation beyond that is limited. Further research where other types of organisations are followed longitudinally is needed to validate the findings. However, similar findings were found in the Swedish municipalities [35].

We had the opportunity to study the implementation of the Stamina model at a hospital department that was about to reorganise their operations. Under these circumstances, there was no room for either a control group or a waiting group design. The re-organisation had to include all employees in the department; thus, choosing another working group outside of the department to be the control group would have created conditions that were too different for the comparison to be really relevant. We therefore chose to follow these groups over time and describe the relationship at baseline and changes over time, as we found it valuable to describe a situation that often arises in real life, although this did not allow a perfectly designed study.

The low number of participants at the fourth time point was mainly due to the coronavirus pandemic. Under those extreme conditions, the new situation and changed work tasks meant that just under a quarter of the participants were able to prioritise completing the process of questionnaires and workshops before the study was completed. We reported the result for the last measurement to show the trend, which, despite the prevailing state of emergency, still showed a continued improvement in the subgroup. The results from the fourth time point were excluded from the statistical analyses. The results indicate that even though specific individuals could not be followed over time, the process and the trends were equally important to show the development in the work environment for the entire group.

## 6. Conclusions

The findings indicate that the HRI measure can be followed over time as a single measure to capture changes in productivity, relational justice and short-term recovery. High HRI-levels seem to be interconnected with low levels of work-related productivity loss, high relational justice and with good short-term recovery. The implementations of the Stamina model, of which the HRI measure constitutes an important part, seem to be a helpful tool to follow-up on the work environment process, minimise production losses due to health and work environment-related issues, and to fulfil the legal provisions.

## Figures and Tables

**Table 1:** (**a**) Descriptive statistics for t1 to t4. (**b**) Descriptive statistics for t1 to t4.

(**a**)					
	**T1 (*n* = 267)** **Mean (SD)**	**T2 (*n* = 214)** **Mean (SD)**	**T3 (*n* = 225)** **Mean (SD)**	**T4 (*n* = 49)** **Mean (SD)**	**Test of Difference (T1 and T3)**
Human Resources Index (HRI)	48.46 (22.47)	53.41 (21.80)	56.65 (21.23)	64.40 (19.87)	*p* < 0.001 †
Relational Justice Index (RJI) *	N.A. **	N.A. **	24.78 (3.70)	26.02 (4.05)	N.A. **
Short-term recovery *	2.61 (1.33)	2.44 (1.20)	2.65 (1.22)	2.80 (1.32)	*p* = 0.872 ‡
(**b**)					
	**T1 (*n* = 267)** **Median (IQR)**	**T2 (*n* = 214)** **Median (IQR)**	**T3 (*n* = 225)** **Median (IQR)**	**T4 (*n* = 49)** **Median (IQR)**	**Test of Difference (T1 and T3)**
Health-related production loss	2 (3)	0 (3)	0 (3)	0 (1)	*p* < 0.001 ‡
Work environment related production loss	2 (3)	2 (5)	0 (4)	0 (3)	*p* < 0.001 ‡

* Reversed items: a high value indicates good relational justice and good recovery. † *t*-test. ‡ Wilcoxon rank sum with continuity correction. t1 was performed during Autumn of 2018, t2 in Spring of 2019, t3 in Autumn of 2019 and t4 in Spring of 2020. ** N.A. = Not applicable due to technical error during data collection.

**Table 2:** Proportion of HRI < 50.0 and proportion of respondents reporting production loss.

	T1 (%)	T2 (%)	T3 (%)	T4 (%)	Test of Difference (T1 and T3)
Human Resources Index HRI < 50.0	55.4	43.0	32.9	26.5	*p* < 0.001 ¥
Health-related production loss	46.0	43.9	38.7	36.7	*p* = 0.552 ¥
Work environment-related production loss	52.4	60.7	49.3	34.7	*p* = 0.118 ¥

¥ 2-sample Z-test for equality of proportions.

**Table 3:** Work environment-related production loss at t1.

	Did Report Work Environment- Related Production Loss (*n* = 127) Mean (SD)	Did Not Report Work Environment-Related Production Loss (*n* = 140) Mean (SD)	*t*-Test Difference	Effect Size Cohen’s d
Human Resources Index (HRI)	40.7 (19.9)	57.9 (22.1)	*p* < 0.001	0.816
Short-term recovery	1.99 (1.86)	2.39 (1.58)	*p* = 0.0586	0.233
Your supervisor considered your viewpoint.	4.11 (0.78)	4.46 (0.72)	*p* < 0.001	0.467
Your supervisor took steps to deal with you in a truthful manner.	3.73 (1.08)	3.94 (1.02)	*p* = 0.1035	0.200
Your supervisor was able to suppress personal biases.	3.56 (1.07)	3.93 (0.93)	*p* = 0.0028	0.370
Your supervisor provided you with timely feedback about the decisions and their implications.	4.42 (0.88)	4.69 (0.61)	*p* = 0.0068	0.334
Your supervisor treated you with kindness and consideration.	4.14 (0.98)	4.44 (0.71)	*p* = 0.0043	0.353
Your supervisor showed concern for your rights as an employee.	N.A. *	N.A. *	N.A. *	N.A. *

Note: Work environment-related production loss: Over the past 7 days, have you experienced any work environment-related problems? 1 = yes, 2 = no. * N.A. = Not applicable due to technical error during data collection.

**Table 4:** Work environment-related production loss at t3.

	Did Report Work Environment-Related Production Loss (*n* = 111) Mean (SD)	Did Not Report Work Environment-Related Production Loss (*n* = 114) Mean (SD)	*t*-Test Difference	Effect Size Cohen’s d
Human Resources Index (HRI)	50.6 (20.4)	62.6 (20.5)	*p* < 0.001	0.516
Relational Justice Index (RJI)	23.8 (3.85)	25.7 (3.31)	*p* < 0.001	0.530
Short-term recovery	2.39 (1.21)	2.91 (1.17)	*p* < 0.001	0.530
Your supervisor considered your viewpoint.	4.22 (0.74)	4.49 (0.68)	*p* = 0.0048	0.380
Your supervisor took steps to deal with you in a truthful manner.	3.37 (1.09)	3.73 (1.06)	*p* = 0.0127	0.335
Your supervisor was able to suppress personal biases.	3.69 (0.96)	3.96 (0.86)	*p* = 0.0272	0.296
Your supervisor provided you with timely feedback about the decisions and their implications.	4.43 (0.86)	4.68 (0.56)	*p* = 0.0202	0.312
Your supervisor treated you with kindness and consideration.	4.15 (0.99)	4.51 (0.64)	*p* = 0.1211	0.280
Your supervisor showed concern for your rights as an employee.	4.05 (0.91)	4.22 (0.72)	*p* = 0.0114	0.340

Note: Work environment-related production loss: Over the past 7 days, have you experienced any work environment-related problems? 1 = yes, 2 = no.

## Data Availability

The data presented in this study are available on request from the corresponding author.
